# Vaccination Viewed by a Potent Enemy of Vaccination

**Published:** 1898-07

**Authors:** Wm. B. Clarke

**Affiliations:** 1223 Senate Ave., N., Indianapolis, Ind.


					﻿VACCINATION VIEWED BY A POTENT ENEMY
OF VACCINATION.
1223 Senate Ave., N.,
Indianapolis, Ind., May 22d, 1898.
My Dear Dr. James:—Your kind letter of recent date is
.at hand.
In my opinion the medical men should wake up on the
vaccination question and learn something more about it
than the mere technique of the operation itself. I have
studied the subject enough to enable me to write thirty arti-
cles on it in the last five years, to take it into our State
society twice, our city society twice, and a national society
twice (and oftener on discussions). I inclose, a few samples,
showing that I have the courage of my convictions. One
of your own papers (Philadelphia Evening Item, May 23d,
1894), did me the honor of quoting largely from one paper,
not one I herewith irvclose. Next Wednesday I read a paper
on “The Treatment of Cancer” before the Kentucky Homoe-
opathic Institute, Frankfort, and shall show how vaccination
is the master cause of cancer that is now fastening such a
•strong hold on the “civilized” world.
If I can find time to-morrow I may make a copy of the
vaccination part of it and send it to you for perusal or such
use as you may see fit.*
* See June number of The Homoeopathic Physician, page 246
Yours fraternally,
Wm. B. Clarke.
				

